# Morphological change of coiled bacterium *Spirosoma linguale* with acquisition of β-lactam resistance

**DOI:** 10.1038/s41598-021-92787-8

**Published:** 2021-06-24

**Authors:** Tomoya Maeda, Hazuki Kotani, Chikara Furusawa

**Affiliations:** 1 RIKEN Center for Biosystems Dynamics Research, 6-2-3 Furuedai, Suita, Osaka 565-0874 Japan; 2 Department of Physics, The University of Tokyo, 7-3-1 Hongo, Tokyo, 113-0033 Japan; 3 Universal Biology Institute, The University of Tokyo, 7-3-1 Hongo, Tokyo, 113-0033 Japan; 4 Present Address: Laboratory of Microbial Physiology, Research Faculty of Agriculture, Hokkaido University, Kita 9, Nishi 9, Kita-ku, Sapporo, Hokkaido 060-8589 Japan

**Keywords:** Evolution, Genetics, Microbiology

## Abstract

*Spirosoma linguale* is a gram-negative, coiled bacterium belonging to the family *Cytophagaceae*. Its coiled morphology is unique in contrast to closely related bacteria belonging to the genus *Spirosoma*, which have a short, rod-shaped morphology. The mechanisms that generate unique cell morphology are still enigmatic. In this study, using the *Spirosoma linguale* ATCC33905 strain, we isolated β-lactam (cefoperazone and amoxicillin)-resistant clones. These clones showed two different cell morphological changes: relatively loosely curved cells or small, horseshoe-shaped cells. Whole-genome resequencing analysis revealed the genetic determinants of β-lactam resistance and changes in cell morphology. The loose-curved clones commonly had mutations in Slin_5958 genes encoding glutamyl-tRNA amidotransferase B subunit, whereas the small, horseshoe-shaped clones commonly had mutations in either Slin_5165 or Slin_5509 encoding pyruvate dehydrogenase (PDH) components. Two clones, CFP1ESL11 and CFL5ESL4, which carried only one mutation in Slin_5958, showed almost perfectly straight, rod-shaped cells in the presence of amoxicillin. This result suggests that penicillin-binding proteins targeted by amoxicillin play an important role in the formation of a coiled morphology in this bacterium. In contrast, supplementation with acetate did not rescue the growth defect and abnormal cell size of the CFP5ESL9 strain, which carried only one mutation in Slin_5509. These results suggest that PDH is involved in cell-size maintenance in this bacterium.

## Introduction

Bacteria show a tremendous diversity of cell morphologies, such as straight rod, curved rod, round, helical, and filamentous^1,2^. It is speculated that ancestral bacteria are rod-shaped, and that coccoid forms evolved from such rod-shaped bacteria^3^. It is also known that some bacteria show unique cell morphologies, such as the star shape of *Stella* species^4^ and coiled shape of *Spirosoma linguale*^5^. Bacterial cell shape can be determined by the spatiotemporal regulation of enzymes involved in peptidoglycan layer synthesis^1,6,7^. The molecular mechanisms for maintenance of cell shape and cell division have been studied extensively in rod-shaped bacteria such as *Escherichia coli*, *Bacillus subtilis*, and a curved-rod shaped *Caulobacter cresentus*^1,6,8^. Since helical shapes of human pathogenic bacteria e.g. *Helicobacter pylori* and *Campylobacter jejuni* are known to provide mechanical advantage for colonization and host interactions in viscous environments^9,10^, enzymes involved in helical shape determination have been also studied. It was suggested that helical shape is maintained in these pathogens by a delicate balance of peptidoglycan length and crosslinking through peptidoglycan modifying enzymes e.g. Pgp1, Pgp2 in *C. jejuni*^11–14^, and several periplasmic peptidoglycan hydrolases and non-enzymatic putative scaffolding proteins e.g. Csd5Csd4, Csd5, Csd6, and CcmA in *H. pylori*^15–20^. However, the molecular basis of how bacteria form more complicated cell shapes is far less well understood.

*S. linguale* ATCC33905 (family *Cytophagaceae*) was isolated from a laboratory water bath as peculiar coiled and horseshoe-shaped cells^5^. This gram-negative bacterium grows aerobically and forms smooth yellow colonies^5^. A previous study reported that the cell width is 0.5–1.0 μm, and the diameter of the outer ring is 1.5–3.0 μm^5^. The genus name *Spirosoma* derives from its coiled body; however, the other known bacteria belonging to *Spirosoma* show normal rod shapes, for example, *S. radiotolerans* KCTC32455, which has the highest sequence similarity to *S. linguale*^21^. This suggests that the unique morphology of *S. linguale* evolved from its normal rod shape.

Inhibition of cell wall synthesis and cell division is a promising approach for understanding how bacteria determine their cell morphology. Treatment of bacterial cells with certain antibiotics, such as β-lactams targeting penicillin-binding proteins (PBPs) and A22 targeting an actin-like MreB protein, cause the formation of abnormal cell shapes in many bacteria, including *E. coli* and *Bacillus subtilis*^22,23^. It was shown that acquisition of resistance to such antibiotics sometimes results in altered cell shape. For example, *E. coli* cells resistant toward mecillinam, which targets PBP2, showed a rounded cell shape^24^. Interestingly, overexpression of a mutated *ftsA* gene resulted in the formation of C-shaped cells in *E. coli*^25^. Since genetic tools for *S. linguale* have not yet been developed, we believe that isolation of β-lactam-resistant clones following whole-genome resequencing can be applied to understand mechanisms for determination of coiled cell morphology in *S. linguale*. In this study, we isolated *S. linguale* clones resistant to either cefoperazone or amoxicillin that showed altered cell morphology. We then conducted a whole-genome resequencing analysis of the resistant clones to identify commonly mutated genes. These results provide insight into the genetic determinants of coiled morphology in *S. linguale*.

## Results

### Isolation of β-lactam-resistant *S. linguale* strains

**Figure 1:**
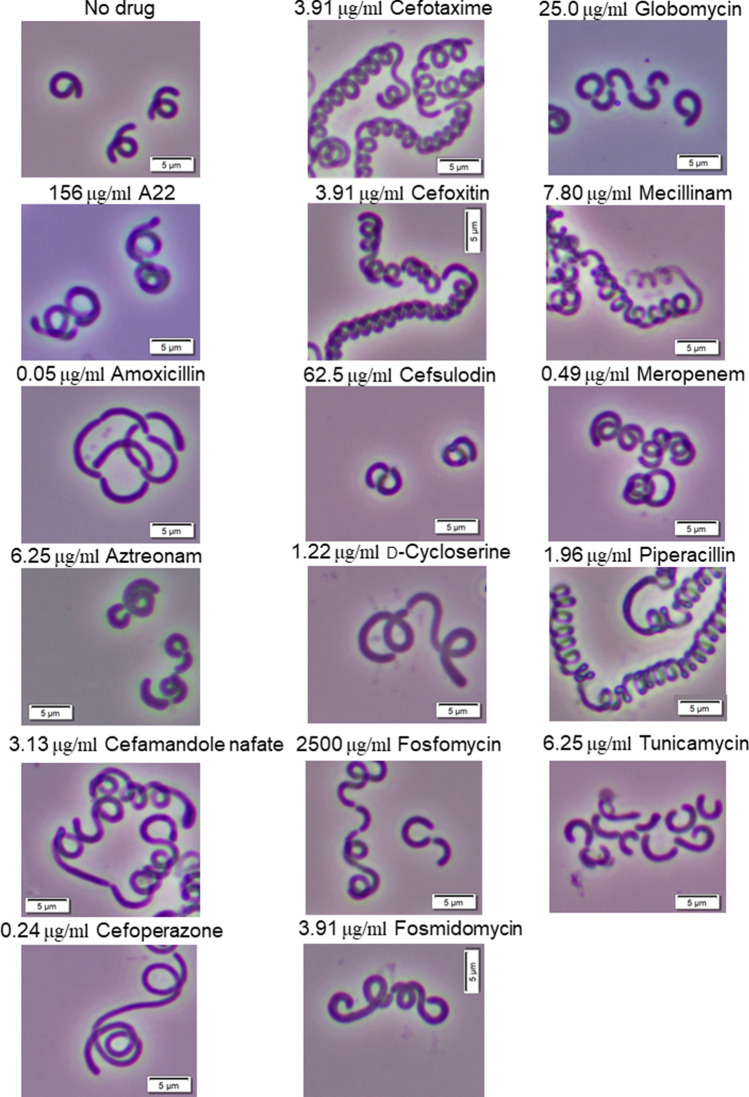
Cell shape of *S. linguale* ATCC33905 strain cells treated with various antibiotics. Microscopy of the ATCC33905 strain when cells were treated with the subinhibitory concentration of each antibiotic indicated above the image. Bar, 5 μm. Cells were cultivated in the stationary phase in *Spirosoma* medium containing the subinhibitory concentration of each antibiotic at 26 °C with shaking. The subinhibitory concentration of each antibiotic is shown alongside the name of antibiotics.

First, to examine the effects of antibiotic treatment on *S. linguale* morphology, we determined the half-maximal inhibitory concentrations (IC_50_ values) of this microorganism to 16 antibiotics, including various β-lactams (Table 1). We observed morphological changes following treatment with sub-inhibitory concentrations of the antibiotics, as shown in Fig. 1. The wild-type *S. linguale* ATCC 33905 strain showed high resistance to fosfomycin, A22, and cefsulodin, and high sensitivity to amoxicillin, meropenem, and cefoperazone (Table 1). A long coil-like morphology was observed when cells were treated with cefamandole nafate, cefotaxime, cefoxitin, mecillinam, and piperacillin (Fig. 1). Amoxicillin treatment caused formation of a relatively loosely curved morphology (Fig. 1). These results indicate that inhibition of peptidoglycan synthesis by β-lactams affects the morphology of *S. linguale*.

**Table 1:** IC_50_ values of 16 antibiotics, including various β-lactams.

Drug	IC_50_ (μg/mL)
A22	312
Amoxicillin	0.10
Aztreonam	12.5
Cefamandole nafate	6.25
Cefoperazone	0.49
Cefotaxime	7.81
Cefoxitin	7.81
Cefsulodin	125
d-Cycloserine	2.44
Fosfomycin	5000
Fosmidomycin	7.81
Globomycin	50.0
Mecillinam	15.6
Meropenem	0.10
Piperacillin	3.91
Tunicamycin	12.5

IC_50_ values (µg mL^−1^) for *S. linguale* ATCC33905 strain are the means from three independent experiments.

**Figure 2:**
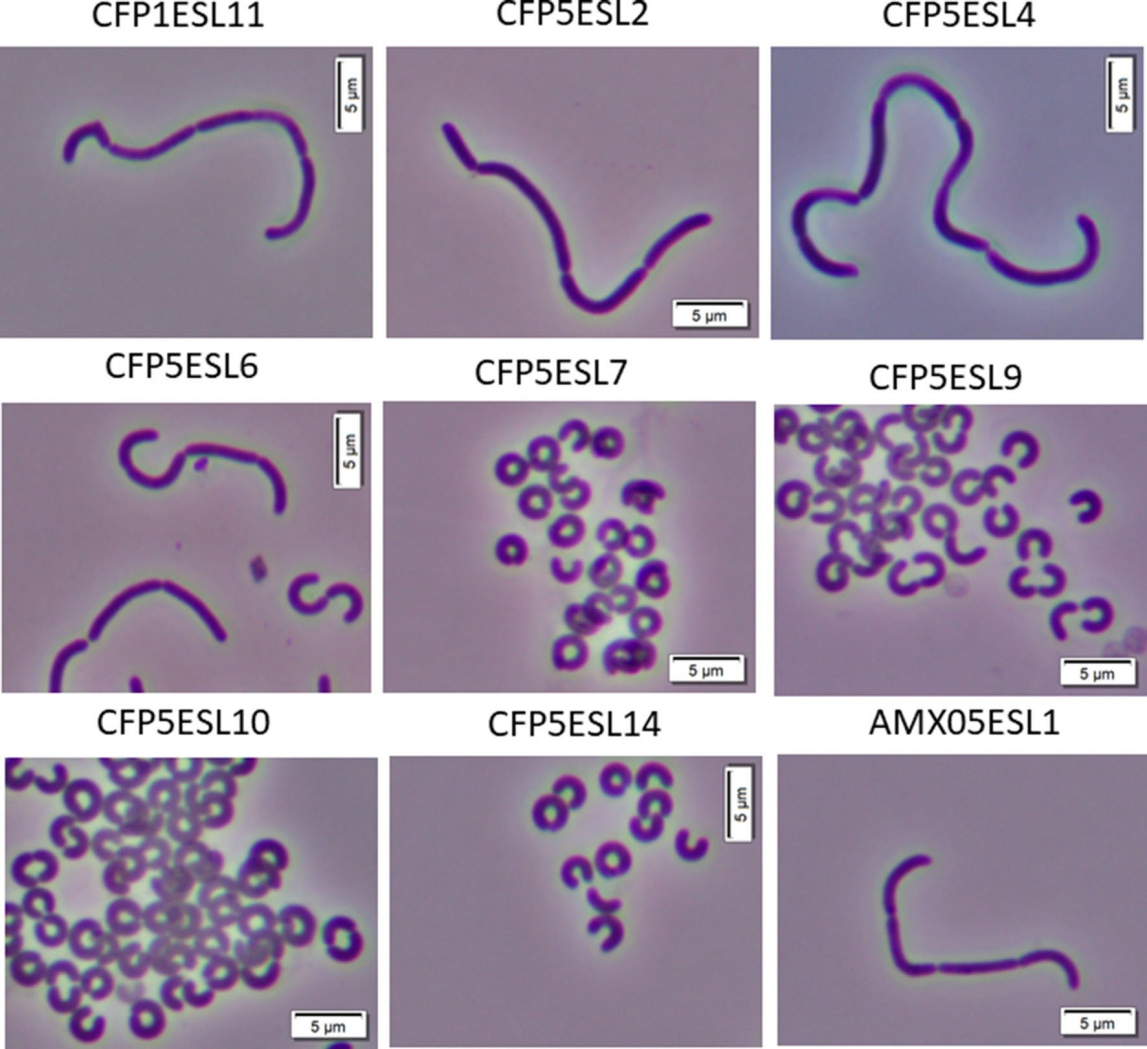
Cell shape of *S. linguale* cefoperazone- and amoxicillin-resistant clones. Microscopy of the cefoperazone-resistant clones (CFP1ESL11, CFP5ESL2, CFP5ESL4, CFP5ESL6, CFP5ESL7, CFP5ESL9, CFP5ESL10, and CFP5ESL14), and the amoxicillin-resistant clone (AMX05ESL1) cells are shown. Bar, 5 μm. Cells were cultivated in the stationary phase in *Spirosoma* medium without antibiotics at 26 °C with shaking.

Next, to obtain *S. linguale* mutant strains showing abnormal morphologies, we selected amoxicillin, cefoperazone, cefotaxime, cefoxitin, mecillinam, and piperacillin-resistant colonies on agar plates containing lethal concentrations of these antibiotics. We successfully isolated 19 amoxicillin-resistant and 32 cefoperazone-resistant colonies. Among these resistant colonies, one amoxicillin-resistant and all 32 cefoperazone-resistant colonies showed two separate abnormal cell morphologies: relatively loose-curve morphology or small, horseshoe-shaped cells. For further analysis, we selected nine cefoperazone-resistant clones and one amoxicillin-resistant clone. The morphologies of the resistant clones without the addition of antibiotics are shown in Fig. 2.

### Whole-genome resequencing analysis of *S. linguale* β-lactam-resistant clones

To identify mutations that contribute to β-lactam resistance and morphological changes in *S. linguale*, we performed a whole-genome resequencing analysis of the ten resistant clones. All resistant strains carried less than three identified mutations, and four strains (CFP1ESL11, CFP1ESL16, CFP5ESL4, and CFP5ESL9) carried only one identified mutation. The list of identified mutations in the resistant strains is listed in Table S1. Among the six strains showing relatively loose-curve morphology, all had acquired mutations in Slin_5958, encoding glutamyl-tRNA amidotransferase B subunit, except for the CFP1ESL16 strain. Three out of the six curved strains also commonly had mutations in Slin_5457, encoding the excinuclease ABC A subunit. Interestingly, CFP1ESL11 and CFP5ESL4 strains carried mutations only in the Slin_5958 gene, and the mutation in the CFP5ESL4 strain was a frameshift mutation. This strongly suggests that inactivation of Slin_5958 results in abnormal morphology. On the other hand, all four strains with small, horseshoe-shaped cells commonly had mutations in genes encoding the pyruvate dehydrogenase (PDH) E1 component—that is, Slin_5509 encoding the α subunit or Slin_5165 encoding the transketolase central region. Among the four strains, the CFP5ESL9 strain carried only one frameshift mutation in Slin_5509. This result suggests that inactivation of pyruvate dehydrogenase results in decreased cell size.

### Involvement of Slin_5958 and PBPs in formation of coiled morphology in *S. linguale*

**Figure 3:**
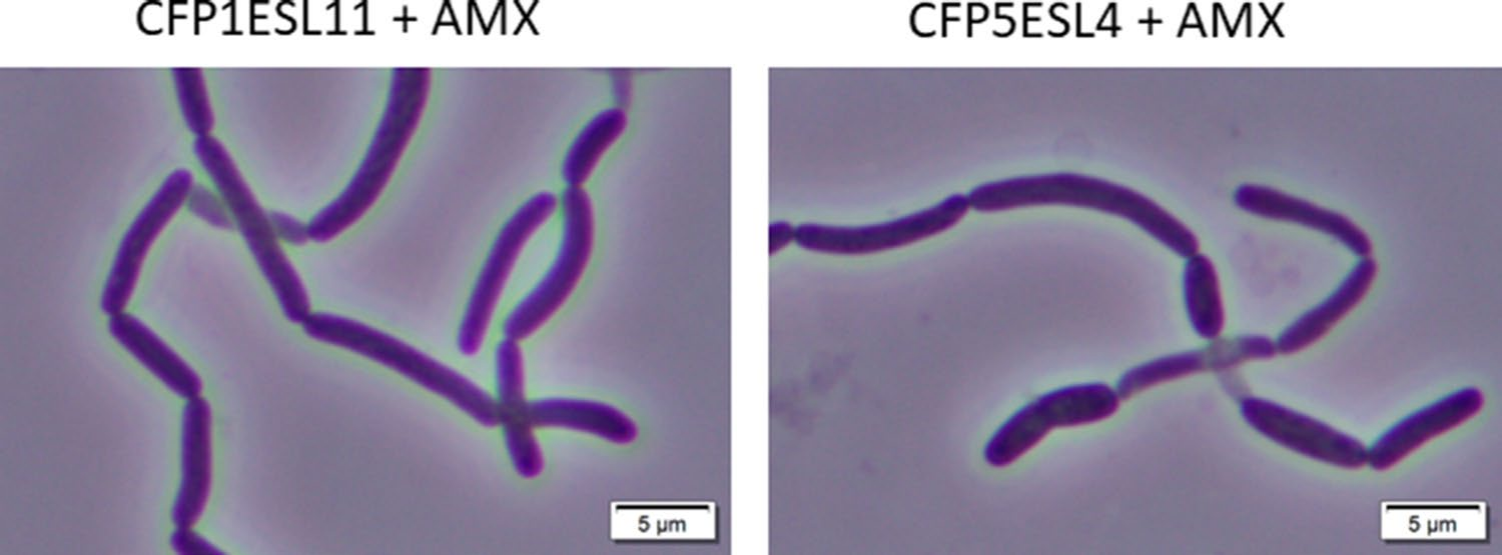
Cell shape of cefoperazone-resistant clones carrying Slin_5958 mutation in the presence of the subinhibitory concentration of amoxicillin. Microscopy of the CFP1ESL11 and CFP5ESL4 strains treated with the subinhibitory concentration (0.78 μg/mL) of amoxicillin. Bar, 5 μm. Cells were cultivated in the stationary phase in *Spirosoma* medium containing a subinhibitory concentration of amoxicillin at 26 °C with shaking.

To examine how the Slin_5958 mutation affects β-lactam resistance, the IC_50_ values of amoxicillin, cefoperazone, cefotaxime, and piperacillin for CFP1ESL11 and CFP5ESL4 strains were determined. Both CFP1ESL11 and CFP5ESL4 strains showed at least a four-fold increase in β-lactam resistance (Table 2). Morphologies of these strains treated with sub-inhibitory concentrations of these four β-lactams were also examined. As shown in Fig. 3, CFP1ESL11 and CFP5ESL4 strains generated straight, rod-shaped cells when treated with amoxicillin. Such straight, rod-shaped cells were not observed when these cells were treated with the other β-lactams. Since the wild-type strain did not generate straight, rod-shaped cells when treated with amoxicillin, a combination of Slin_5958 inactivation and inhibition of the target PBPs of amoxicillin resulted in the formation of straight, rod-shaped cells.

**Table 2:** IC_50_ values of β-lactams for β-lactam-resistant strains.

Drug	IC_50_ (μg/mL)				
	WT	CFP1ESL11	CFP5ESL4	CFP5ESL7	CFP5ESL9
Cefoperazone	0.49	15.6	15.6	7.81	7.81
Amoxicillin	0.10	1.56	1.56	0.39	0.39
Piperacillin	3.91	31.3	31.3	15.6	15.6
Cefotaxime	7.81	31.3	31.3	15.6	15.6

IC_50_ values (µg mL^−1^) for the wild-type strain and the four cefoperazone-resistant clones (CFP1ESL11, CFP5ESL4, CFP5ESL7, and CFP5ESL9) are the means of three independent experiments.

### Inactivation of PDH E1 components results in the formation of small *S. linguale* cells

**Figure 4:**
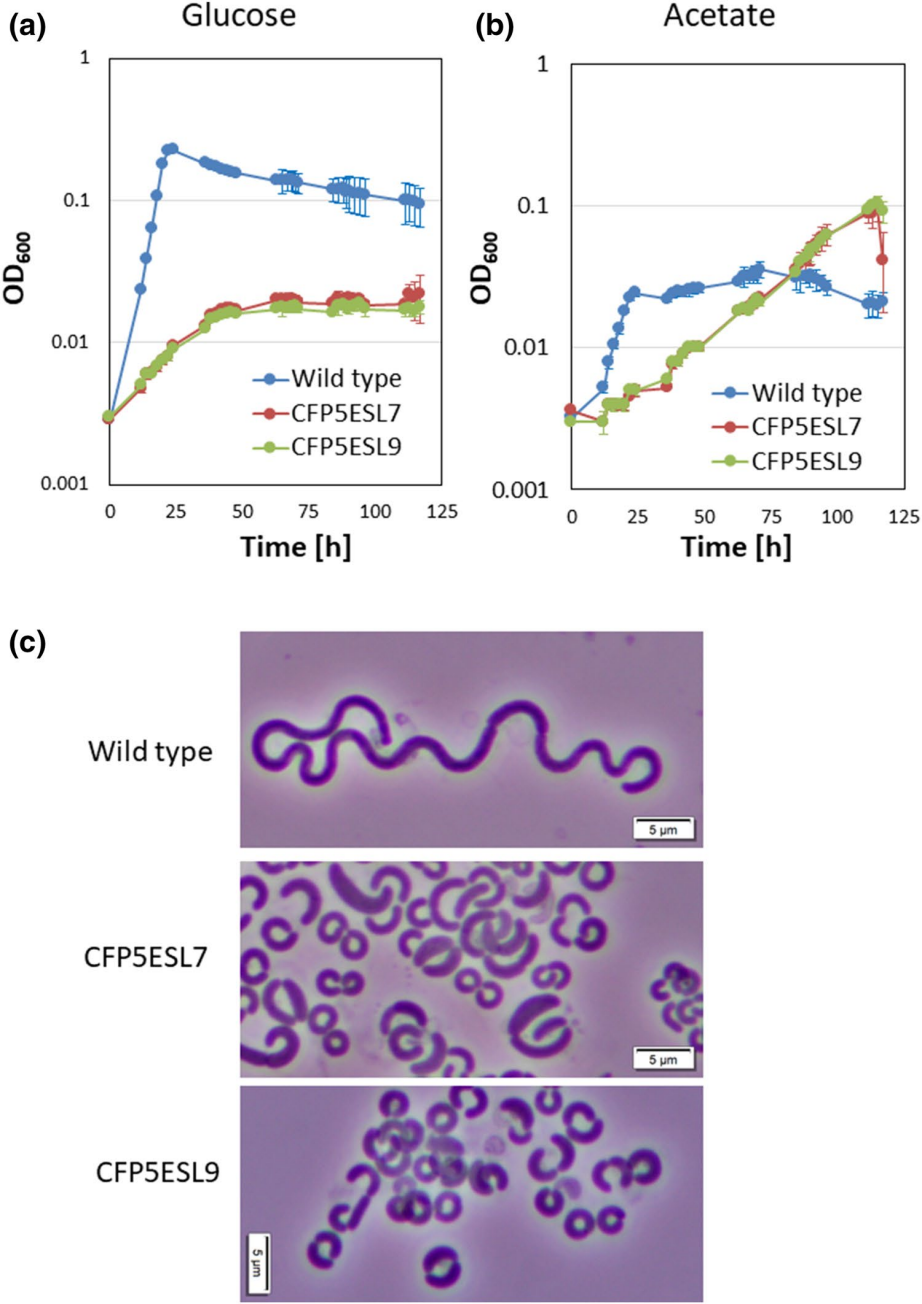
Growth of wild-type and cefoperazone-resistant clones carrying PDHc-deficient mutations. (**a**,**b**) Growth of the wild-type (ATCC33905) and cefoperazone-resistant clones (CFP5ESL7 carrying Slin_5165 encoding the PDHc transketolase central region and CFP5ESL9 carrying only one mutation in Slin_5509 encoding PDH E1 α subunit) are shown. Blue: wild type, red: CFP5ESL7, green: CFP5ESL9. Cells were grown on *Spirosoma* medium containing either 0.1% (wt vol^-1^) glucose (**a**) or 0.1% acetate (**b**) at 26 °C with shaking. Growth was monitored by measuring OD_600_. Each growth measurement was performed three times, starting from independent cultures. Error bars represent standard deviation. (**c**) Microscopy of the wild-type, CFP5ESL7, and CFP5ESL9 strains grown on *Spirosoma* medium supplemented with 0.1% acetate at 26 °C with shaking are shown. Bar, 5 μm.

To investigate the effect of the Slin_5509 mutation on β-lactam resistance, we first determined the IC_50_ values of amoxicillin, cefoperazone, cefotaxime, and piperacillin for CFP5ESL7 and CFP5ESL9 strains. Both of these strains showed at least a two-fold increase in β-lactam resistance (Table 2). Since CFP5ESL9 strains carry only one mutation in Slin_5509, which encodes the PDH E1 α subunit, a decrease in central metabolism may affect both β-lactam resistance and cell size in *S. linguale*. Both CFP5ESL7 and CFP5ESL9 strains showed severe growth defects in *Spirosoma* medium (Fig. 4a). The growth rates of the wild-type, CFP5ESL7, and CFP5ESL9 strains in this medium supplemented with 0.1% glucose were 2.57 × 10^–1^, 4.63 × 10^–2^, and 4.90 × 10^–2^ h^–1^ respectively. Both CFP5ESL7 and CFP5ESL9 strains showed approximately a five-fold decrease in final OD_600_ values (Fig. 4a). Since PDHc plays a major role in acetyl-CoA supply, we next examined whether the increase in acetyl-CoA supply in the PDHc mutant restored cell size. To increase acetyl-CoA levels via an alternative reaction (for example, acetyl-CoA synthetase), acetate was added to the medium. The reduced growth rate was not restored by supplementation with 0.1% acetate, though supplementation with 0.1% acetate improved the final OD_600_ value of both CFP5ESL7 and CFP5ESL9 strains (Fig. 4b). Acetate supplementation also did not rescue the cell sizes of CFP5ESL7 or CFP5ESL9, except for a few minor cells showing relatively loose-curve morphology (Fig. 4c). Interestingly, acetate supplementation resulted in the formation of an elongated morphology in the wild-type strain (Fig. 4c). These results suggest that the supply shortage of acetyl-CoA by PDHc mutation is not a trigger for the formation of small *S. linguale* cells. This observation implies a role for PDHc in regulation of the cell cycle, in addition to acetyl-CoA production.

## Discussion

In this study, we isolated two different β-lactam-resistant *S. linguale* strains showing either relatively loosely curved cells or small, horseshoe-shaped cells. Whole genome resequencing analysis identified two β-lactam resistance-conferring mutations in Slin_5958, encoding glutamyl-tRNA amidotransferase B subunit, and in genes encoding PDH E1 components. Although our attempts to develop genome-editing technology for *S. linguale* failed, analysis of the resistant strains carrying only one mutation in either Slin_5958 or Slin_5509 suggests involvement of these mutations in both β-lactam resistance and morphological changes. To investigate the effect of these mutations on cell division and/or chromosome segregation, localization of septa and nucleoids will be investigated as future works.

Both CFP1ESL11 and CFP5ESL4 strains carried only one mutation in the Slin_5958 gene, and treatment of these cells with sub-inhibitory concentrations of amoxicillin resulted in formation of nearly straight, rod-shaped cells (Fig. 3). Glutamyl-tRNA amidotransferase catalyzes the formation of glutamine-tRNA by the transamidation of misacylated glutamate-tRNA via amidolysis of glutamine^26^. Since *S. linguale* have glutaminyl-tRNA synthetase encoded by Slin_2363, the activity of glutamyl-tRNA amidotransferase can be replaced by the glutaminyl-tRNA synthetase. This suggests that Slin_5958 encoding the B subunit of glutamyl‑tRNA amidotransferase is non-essential. Since *S. linguale* has both pathways to form glutaminyl-tRNA, the glutamyl-tRNA amidotransferase activity may function as a feedback control mechanism to regulate intracellular concentrations of glutamate and glutamine. Interestingly, a mutation in the gene encoding the glutamyl-tRNA amidotransferase B subunit of rice has been reported to cause a non-lethal phenotype of smaller root meristem and shorter root cell length^27^. It was speculated that the decrease in activity of glutamyl-tRNA amidotransferase in the root cells of rice resulted in decreased ATP synthesis, and thereby abnormal growth and morphology^27^. Similar to the rice cells, the mutation in Slin_5958 may cause changes in ATP synthesis and cell morphology. It is also possible that the amidotransferase activity of Slin_5958 itself is directly involved in PG synthesis. It is known that amoxicillin has high specificity to PBP4, which has both DD-endopeptidase and DD-carboxypeptidase activities in *E. coli*^28^. Among the four β-lactams (amoxicillin, cefoperazone, cefotaxime, and piperacillin), only amoxicillin treatment resulted in formation of straight, rod-shaped cells among Slin_5958-mutated cells (Fig. 3). These results suggest that amoxicillin-targeted PBP is involved in the formation of a coiled morphology in *S. linguale*. The genome sequence of *S. linguale* revealed that this bacterium has eight PBPs that are summarized in Table 3. Since amoxicillin specifically targets PBP4 in *E. coli*^28^, the PBP4 homologs encoded by Slin_2717 and Slin_5444 may also be targeted by amoxicillin. Our preliminary investigation suggested that *S. lingual*e genome does not carry homologs of enzymes which are important for the maintenance of the helical shapes in *H. pylori* and *C. jejuni* i.e. Pgp1, Pgp2, Csd5Csd4, Csd5, Csd6, and CcmA. This result suggested that the determinants of the coiled shape in *S. linguale* are different from that of known helical bacteria such as *H. pylori* and *C. jejuni*. Therefore, the genetic determinants of this unique morphology in *S. linguale* other than Slin_5958 and amoxicillin targeted PBPs are unknown.

**Table 3:** Genes encoding PBPs in *S. linguale* and putative β-lactam antibiotics potentially targeting the PBPs.

Gene	Slin_3074	Slin_3220	Slin_5076	Slin_3202	Slin_4738	Slin_2717	Slin_5444	Slin_1280
Annotation	PBP1A	PBP1A	PBP1C	PBP2	PBP3	PBP4	PBP4	FtsW/RodA/SpoVE family protein
Targetting drug*	Cefsulodin	Cefsulodin		Mecillinam	Aztreonam	Amoxicillin	Amoxicillin	
	Cefoperazone	Cefoperazone		Cefoperazone	Piperacillin			
	Cefamandole nafate	Cefamandole nafate			Cefotaxime			
					Cefoperazone			
					Cefamandole nafate			

Genes encoding PBPs in *S. linguale* ATCC33905 strain are listed based on BLAST search.

*Putative β-lactam antibiotics potentially targeting the PBPs of *S. linguale* are listed based on the results of affinity assays for *E. coli* PBPs by previous studies^28,37–41^.

It was found that inactivation of PDHc in *S. linguale* resulted in the formation of small cells (Fig. 4). In both *E. coli* and *B. subtilis*, it has been reported that mutations in genes encoding central metabolism, including acetate metabolism, can suppress temperature sensitivity of replication-defective mutant strains^29–32^. It has also been reported that the PDH E1 α subunit is involved in nutrient-dependent control of Z-ring formation in *B. subtilis* through its association with nucleoid^33^. These results suggest an important role for PDHc in regulation of the cell cycle in *S. linguale*. Inactivation of PDHc in *S. linguale* was not lethal, although the growth rate decreased significantly. According to the KEGG database, *S. linguale* may have an alternative pathway to synthesize acetyl-CoA composed of thiamine pyrophosphate (TPP) domain-containing pyruvate oxidase encoded by Slin_6370 and acetyl-CoA synthetase encoded by Slin_2042. It was shown that the TPP-domain-containing pyruvate oxidase in *E. coli* converts pyruvate to acetate and CO_2_^34^. In *E. coli*, it is also known that synthesized acetate can be converted to acetyl-CoA by acetyl-CoA synthetase^35^. Since *S. linguale* contains orthologs of both TPP-domain-containing pyruvate oxidase and acetyl-CoA synthetase, acetyl-CoA can thus be supplied to the *S. linguale* PDHc mutant strains. This might be the basis for the morphological changes observed in the PDH mutant.

## Methods

### Bacterial strains and growth media

*S. linguale* strain ATCC33905 was obtained from the American Type Culture Collection (Manassas, VA, USA). *S. linguale* cells were cultured in the medium designated by ATCC (*Spirosoma* medium, ATCC medium 1290) containing 1 g/L glucose, 1 g/L peptone, and 1 g/L yeast extract (pH 6.8–7.0). When cells were grown on agar plates, 15 g/L agar was added to *Spirosoma* medium. *S. linguale* cells were cultivated in *Spirosoma* liquid medium with shaking at 150 rotations/min at 26 °C.

### Determination of half-maximal inhibitory concentration (IC_50_)

Since minimum inhibitory concentration (MIC) is determined by identifying the lowest antimicrobial concentration that completely inhibited growth, however, some resistant strains carrying mutations in genes encoding pyruvate dehydrogenase (PDH) components already showed severe growth defects in the absence of antibiotics. Therefore, we measured IC_50_ rather than MIC. To determine IC_50_ values, serial dilutions of antibiotics were prepared in 96-well microplates containing 200 µL of *Spirosoma* medium per well and a two-fold drug gradient in 22 dilution steps were used. Drug gradients depended on the maximum fold changes in IC_50_ values for the evolved strains against the corresponding drugs. To start the IC_50_ measurement, *S. linguale* cells were inoculated from frozen glycerol stock into *Spirosoma* medium and cultivated for 48 h at 26 °C with shaking. Cell growth was monitored by measuring the OD_600_ of each well using a 1420 ARVO microplate reader (PerkinElmer Inc.). The OD_600_ values of the precultures were measured, and precultured cells, calculated to have an initial OD_600_ value of 0.01, were inoculated into each well (5 μL of diluted culture into 200 μL of medium per well) of the 96-well microplates and cultivated with agitation at 900 rotations/min at 26 °C. After 48 h of cultivation, the OD_600_ of the cultures was measured. The IC_50_ was defined as the lowest concentration of antibiotics that reduced the final OD_600_ value by 50% when cells were grown without antibiotics. Each IC_50_ measurement was performed three times, starting from independent cultures.

### Isolation of β-lactam-resistant *S. linguale* strains

*S. linguale* ATCC33905 strain was first cultivated in the *Spirosoma* liquid medium at 26 °C for 48 h. Then, 1 mL of the culture was propagated on *Spirosoma* agar plates containing either 1.5 × 10^–1^ μg/mL amoxicillin, 1.0 μg/mL cefoperazone, or 5.0 μg/mL cefoperazone, and incubated until single colonies appeared. Single clones were isolated on the *Spirosoma* agar plate without antibiotics and used for further analysis.

### Microscopic observations

To examine cell morphology of the wild-type and resistant strains, single-cell images were acquired using an upright microscope (BX53, Olympus, Tokyo, Japan).

### Whole-genome sequencing

Genomic DNA was prepared as follows: strains were grown in 5 mL of *Spirosoma* medium at 26 °C for 48 h. Cells were collected by centrifugation at 4 °C and 20,000×*g* for 5 min, and pelleted cells were stored at − 80 °C before genomic DNA purification. Genomic DNA was isolated and purified using a DNeasy Blood and Tissue Kit (Qiagen, Hilden, Germany) according to the manufacturer's instructions. The quantity and purity of the genomic DNA were determined by measuring the absorbance at 260 nm and calculating the ratio of absorbance at 260 and 280 nm (A260/280) using a NanoDrop ND-2000 spectrophotometer. The A260/280 values of all samples were greater than 1.7. Purified genomic DNA was stored at − 30 °C until use. The genome sequences were analyzed using the Illumina MiSeq System. A 150-bp paired-end library was generated according to the Illumina protocol and sequenced using Illumina MiSeq. In this study, 11 samples (10 evolved strains and the parent strain) with different barcodes were mixed and sequenced, resulting in approximately 140-fold coverage, on average. The potential nucleotide differences were validated using BRESEQ version 0.28^36^.

## Supplementary Information

Supplementary Table S1.

## Data Availability

The raw sequence data of genome sequence analyses are available in the DDBJ Sequence Read Archive under accession number DRA011434.
